# Temperature-induced physiological stress and reproductive characteristics of the migratory seahorse *Hippocampus erectus* during a thermal stress simulation

**DOI:** 10.1242/bio.032888

**Published:** 2018-05-15

**Authors:** Geng Qin, Cara Johnson, Yuan Zhang, Huixian Zhang, Jianping Yin, Glen Miller, Ralph G. Turingan, Eric Guisbert, Qiang Lin

**Affiliations:** 1 CAS Key Laboratory of Tropical Marine Bio-resources and Ecology, South China Sea Institute of Oceanology, Chinese Academy of Sciences, No.164 Xingangxi Rd, Haizhu District, Guangzhou 510301, China; 2 University of Chinese Academy of Sciences, 19A Yuquan Rd, Shijingshan District, Beijing 100049, China; 3 Department of Biological Science, Florida Institute of Technology, 150 W. University Blvd, Melbourne, FL 32901, USA

**Keywords:** Seahorse, Temperature, Reproduction, Reproductive endocrine regulation, Migration

## Abstract

Inshore-offshore migration occurs frequently in seahorse species, either because of prey opportunities or because they are driven by reproduction, and variations in water temperature may dramatically change migratory seahorse behavior and physiology. The present study investigated the behavioral and physiological responses of the lined seahorse *Hippocampus erectus* under thermal stress and evaluated the potential effects of different temperatures on its reproduction. The results showed that the thermal tolerance of the seahorses was time dependent. Acute thermal stress (30°C, 2–10 h) increased the basal metabolic rate (breathing rate) and the expression of stress response genes (*Hsp* genes) significantly and further stimulated seahorse appetite. Chronic thermal treatment (30°C, 4 weeks) led to a persistently higher basal metabolic rate, higher stress response gene expression and higher mortality rates, indicating that the seahorses could not acclimate to chronic thermal stress and might experience massive mortality rates due to excessively high basal metabolic rates and stress damage. Additionally, no significant negative effects on gonad development or reproductive endocrine regulation genes were observed in response to chronic thermal stress, suggesting that seahorse reproductive behavior could adapt to higher-temperature conditions during migration and within seahorse breeding grounds. In conclusion, this simulation experiment indicates that temperature variations during inshore-offshore migration have no effect on reproduction, but promote significantly high basal metabolic rates and stress responses. Therefore, we suggest that the observed high tolerance of seahorse reproduction is in line with the inshore-offshore reproductive migration pattern of lined seahorses.

This article has an associated First Person interview with the first author of the paper.

## Abstract (precis)

**Summary:** We found that seahorse reproduction could adapt to higher-temperature conditions, but seahorses might experience massive mortality rates due to high basal metabolic rates and stress damage under chronic thermal stress.

## INTRODUCTION

Seahorses (genus *Hippocampus*) were previously regarded to have small home ranges and to be a sedentary fish species distributed in patchy areas characterized by shelters such as sea grass, mangroves, and coral reefs (Curtis and Vincent, 2006; Caldwell and Vincent, 2013). Little genetic differentiation and high gene flow have been found among distantly geographically located populations in several seahorse species (Zhang et al., 2014; Boehm et al., 2015), suggesting that seahorses might frequently travel among patchy habitat areas and even across long distances to faraway habitats. The most recent research on this topic, based on several years of investigative data, revealed that the temperate seahorse *Hippocampus mohnikei* experiences seasonal inshore-offshore migration yearly (Qin et al., 2017), and the lined seahorse *Hippocampus erectus* emerges in coastal areas during the warm season and then migrates offshore when the seawater cools (Teixeira and Musick, 2001; Boehm et al., 2015). Circumstantial evidence also implies the existence of seasonal migration phenomena in other seahorse species (Foster and Vincent, 2004).

Seahorses may migrate for many reasons, such as habit, diet, or even human activities along local coasts. In migratory *H. mohnikei*, temporal and spatial abundances align with temperature changes (Qin et al., 2017), with the seahorses actively migrating into shallow coastal seawater areas where the temperature is approximately 25–28°C during warm seasons (Qin et al., 2017). On the other hand, in the case of passive and slow rafting transport, seahorses might be subject to insufferable risks because of exposure to high temperatures (Teske et al., 2007). Therefore, seawater temperature plays an essential role in the identification of seahorse behavioral and physiological characteristics during migration.

Reproduction in teleosts is generally considered to be highly sensitive to thermal stress (Pörtner and Farrell, 2008); a small but chronic change in water temperature might dramatically affect reproductive traits in some ﬁsh (Lin et al., 2006; Zucchetta et al., 2012). Seahorses exhibit a unique ovoviviparous reproduction strategy in which male seahorses hatch embryos through their brood pouches and gestation times are mainly affected by water temperatures (Foster and Vincent, 2004; Lin et al., 2007). In the wild, *H. mohnikei* exhibit high efficiency in mating and hatching during warm seasons (Qin et al., 2017), and pregnant *H. erectus* seahorses are also more frequently observed in summer (Teixeira and Musick, 2001; Baum et al., 2003). The temperature range that an organism can tolerate is expected to narrow with prolongation of the duration of thermal challenge, suggesting that a trade-off in reproductive behaviors exists between tolerance to acute and chronic exposure to thermal stress (Rezende et al., 2011), and the gonad development and gestation time of seahorses are directly affected by the total accumulated temperature (Lin et al., 2007). However, few studies have examined the changes in physiological characteristics or the molecular regulatory mechanisms involved in reproduction under temperature variations. Therefore, given that seahorses might suffer from both the acute thermal stress of high seawater temperature during migration and chronic thermal stress at their breeding grounds, it should be investigated whether seahorses can tolerate a wide temperature range in a short period of time or exhibit greater tolerance to chronic thermal stress.

The functions of some genes reflect stresses in teleosts, and their active regulation can indicate the organism's behavioral and physiological responses (Miranda et al., 2013; Zhang et al., 2018). For example, the members of the *Hsp* gene family (heat shock proteins, such as *Hsp70* and *Hsp60*), which are strongly up-regulated to protect cells from thermal stress by inhibiting apoptosis and repairing proteins, are commonly considered useful genetic markers for evaluating stress response levels (Sorger and Pelham, 1988; Feder and Hofmann, 1999). The *leptin* gene functions to balance energy expenses by inhibiting hunger, and higher *leptin* levels in fasting seahorses are involved in anorexic behavioral responses (Schwartz et al., 2000; Pan et al., 2014; Zhang et al., 2016). In addition, thermal stress can disrupt the function of the fish reproductive brain–pituitary–gonad axis and alter gonad development and spawning behavior (Miranda et al., 2013; Shahjahan et al., 2017). Gonadotropin-releasing hormone (GnRH) and follicle-stimulating hormone (FSH) are critical in the endocrine control of reproduction, promoting the synthesis of sex steroids and regulating gametogenesis (Levavi-Sivan et al., 2010).

The lined seahorse *H. erectus* is a eurythermal species that mainly occurs from the Gulf of Mexico to Nova Scotia in water temperatures ranging from 5 to 28°C (Teixeira and Musick, 2001; Lourie et al., 2016). The effects of temperature on the growth and metabolism of juvenile seahorses have been studied (Lin et al., 2009; Mascaró et al., 2016). However, little is known about the physiological and molecular responses of seahorses to thermal stress because the number of available adult seahorses is small, and rearing techniques have been developed only for few seahorse species (Lin et al., 2016). Through thermal stress simulation, the present study aimed to examine the changes in the behavioral and physiological responses of adult seahorses to thermal stress, especially the changes in their metabolic rates and reproduction, and the findings of this work reflect seahorse responses in the wild to water temperature changes during long- or short-distance migrations.

## RESULTS

### Behavioral and physiological responses to acute thermal stress

**Fig. 1.**
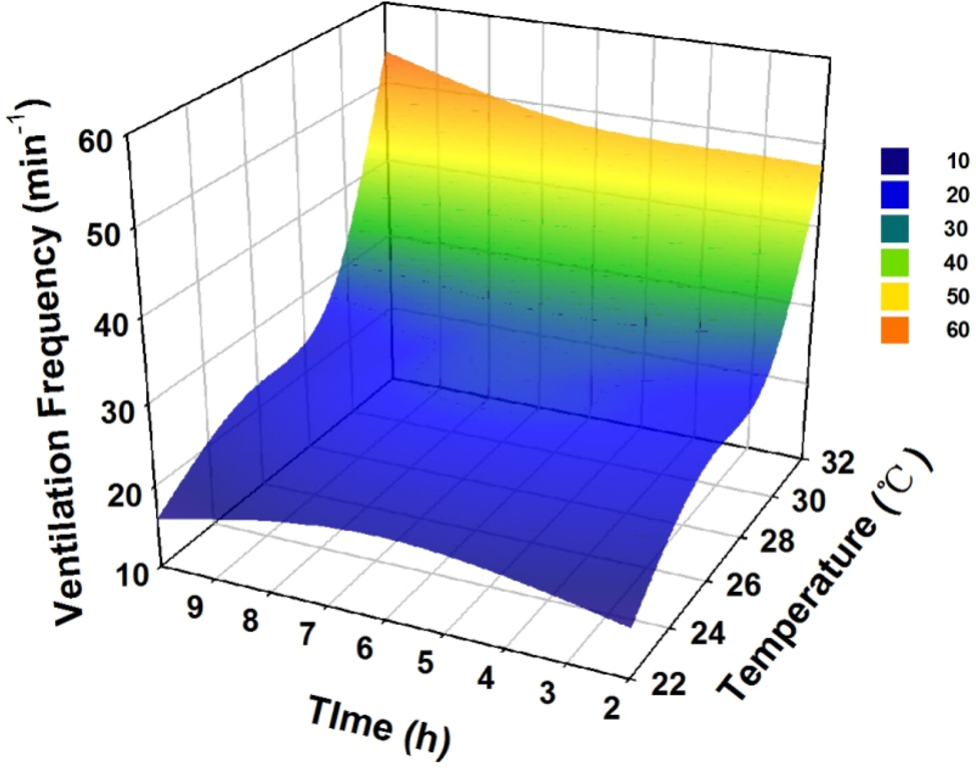
**Seahorse ventilation rate in response to acute thermal stress over 10** **h, when the temperature was increased from 22°C to 32°C.**

**Fig. 2.**
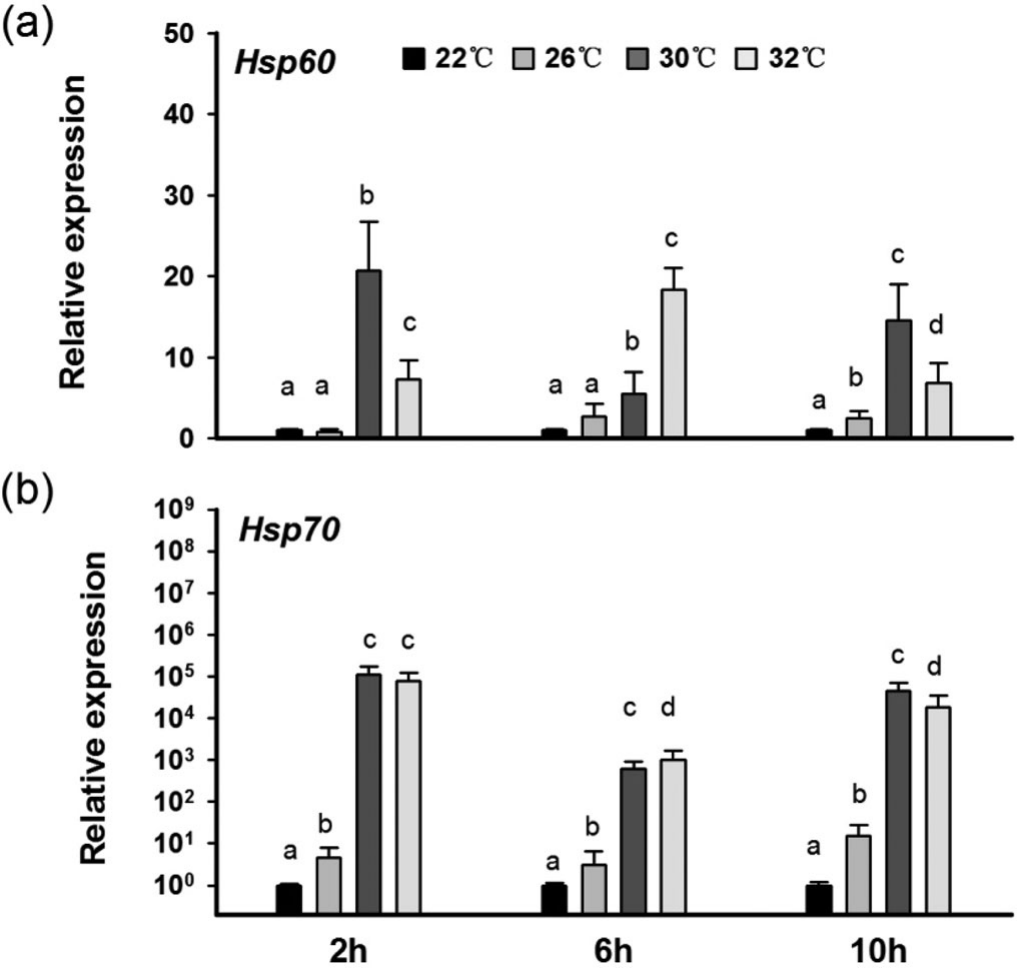
***Hsp60* and *Hsp70* expression in seahorse livers in response to acute thermal stress.** (A) *Hsp60* gene expression. (B) *Hsp70* gene expression. Different letters indicate significant differences between the different groups.

The basal metabolic rates of the adult seahorses were sensitive to an acute rise in temperature. The ventilation rate increased significantly from the 22°C to 26°C (*P*<0.05) or 30°C (*P*<0.05) treatments, while no significant difference in the ventilation rate was found between the 26°C treatment and the 30°C treatment. When exposed to a temperature of 32°C, the ventilation rate increased constantly from 40 min^−1^ at 2 h to 60 min^−1^ at 10 h, leading to death (Fig. 1). Acute thermal stress also increased *Hsp60* and *Hsp70* gene expressions in seahorses, and *Hsp70* expression increased much faster and in a larger range in response to thermal stress. *Hsp70* expression in the seahorses’ livers increased dramatically in both the 30°C treatment (*P*<0.01) and the 32°C treatment (*P*<0.01). *Hsp60* expression at 30°C increased 20-fold at 2 h and then decreased compared with the control group and *Hsp60* expression peaked at 6 h at 32°C. In contrast, the gene expression of *Hsp60* and *Hsp70* in seahorses increased slightly at 26°C (Fig. 2A,B).

### Behavioral and physiological responses to chronic thermal stress

**Fig. 3.**
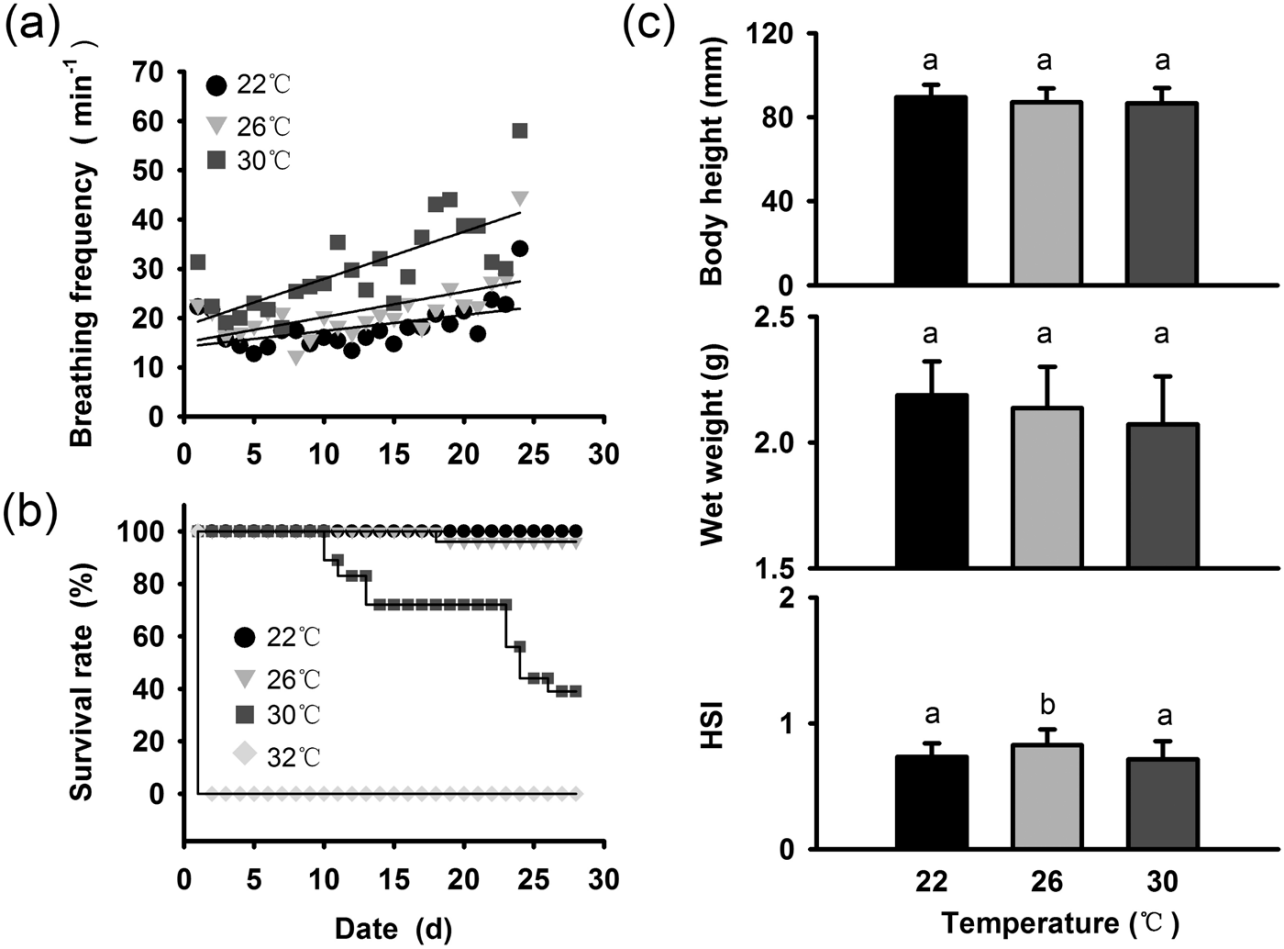
**Ventilation rate (A), mortality rate (B) and growth (C) of seahorses in response to chronic thermal stress.**

Chronic thermal stress gradually increased the seahorses’ basal metabolic rate. The ventilation rate of seahorses held at 22°C remained below 20 min^−1^ most of the time. The ventilation rate of the 26°C seahorses (28 min^−1^) was always higher than that of the 22°C seahorses (*P*<0.05). The ventilation rate of the 30°C seahorses increased to more than 60 min^−1^ in the fourth week, which was much higher than the ventilation rate of the 26°C seahorses (*P*<0.05) or 22°C seahorses (*P*<0.05) (Fig. 3A). The seahorses held at 30°C were visibly agitated and stressed. Few hitching seahorses were observed at 30°C (46%) compared to the 22°C (70%) and 26°C (62%) groups. 37% seahorses kept swimming at 30°C, while only 25% and 17% seahorses were swimming at 22°C and 26°C, respectively (Fig. S1). Thermal stress caused significant mortality rates in seahorses. All seahorses held at 32°C died on the first day, seahorses at 30°C began to die in the second week and 61% seahorses died at the end of the experiment. Only 4% seahorses died at 26°C treatments and no seahorses died at 22°C (Fig. 3B). The growth rates measured in terms of body height and wet weight were not significantly different between the different temperature treatments, but the seahorse hepatosomatic index (HSI) was much higher in the 26°C treatment than in the 22°C and 30°C treatments (Fig. 3C).

**Fig. 4.**
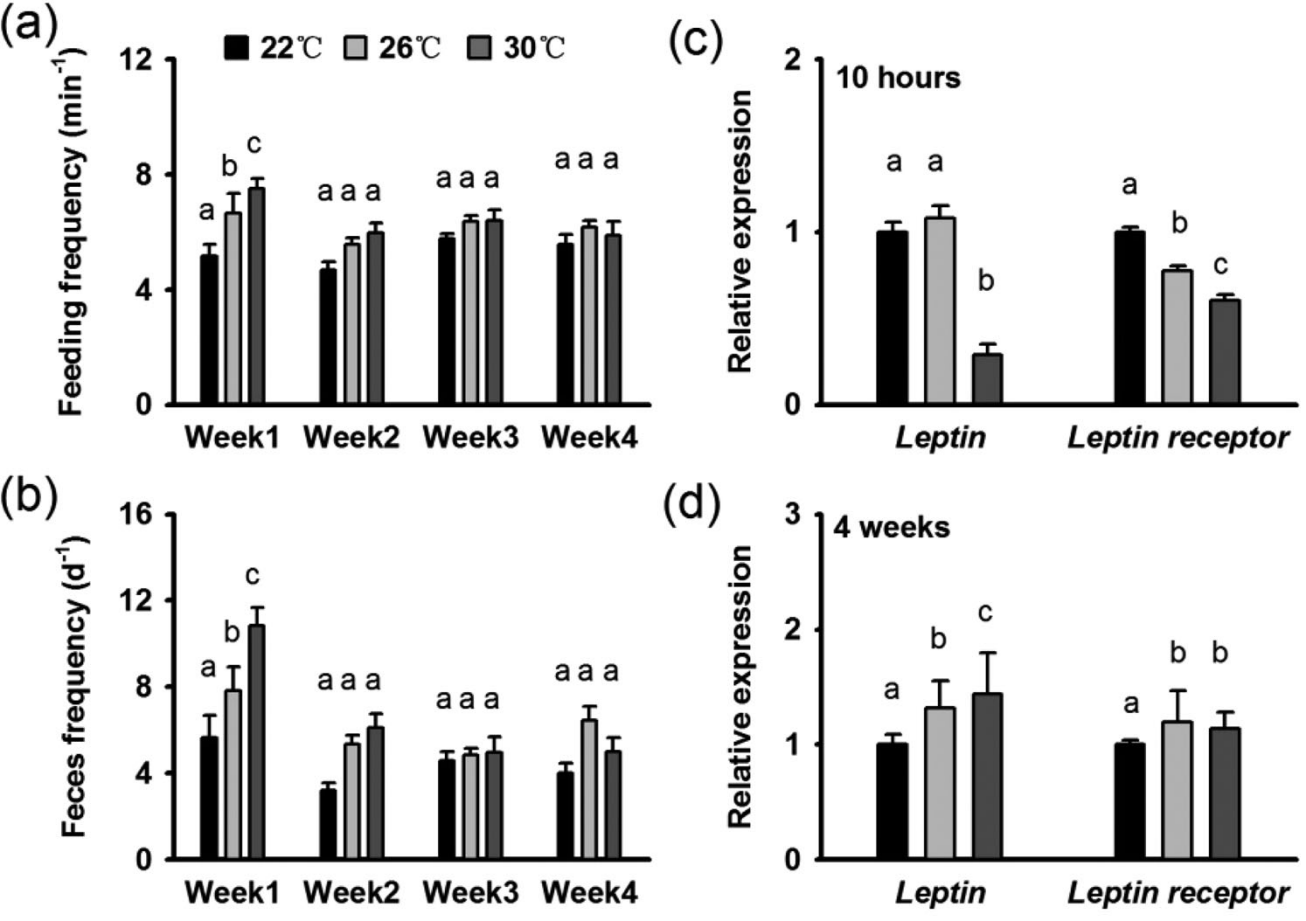
**Feeding behavior and related gene expression of seahorses in response to thermal stress.** (A,B) Feeding frequency (A) and defecation frequency (B) over 4 weeks of thermal stress. (C,D) *leptin* expression in the seahorses’ brains after 10 h (C) or 4 weeks (D) of thermal stress. Different letters indicate significant differences between different groups.

The feeding and defecation frequencies of the seahorses exposed to 30°C improved significantly in the first week (*P*<0.05) and then recovered to normal levels (Fig. 4A,B). *leptin* and *leptin* *receptor* mRNA expression in the seahorses' brains was significantly inhibited under exposure to 30°C during the acute experiment but showed no difference compared with the 26°C seahorses during chronic thermal stress (Fig. 4C,D).

**Fig. 5.**
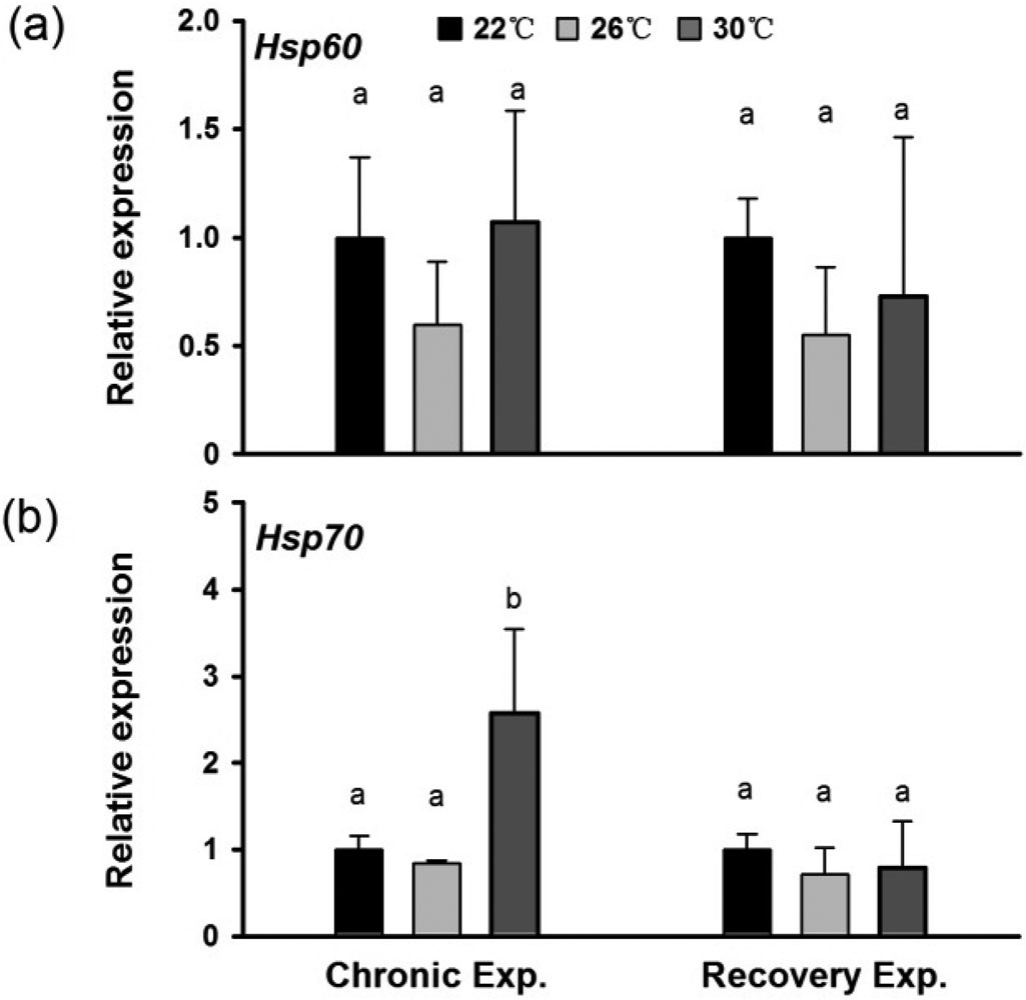
***Hsp* gene expression in response to chronic thermal stress (4 weeks of thermal stress) and room-temperature acclimatization (4 additional weeks of normal temperature acclimation).** (A) *Hsp60* gene expression. (B) *Hsp70* gene expression. Different letters indicate significant differences between different groups.

The expression of *Hsp70* mRNA in the seahorses’ livers in response to chronic thermal stress was increased significantly by less than 10-fold (*P*<0.05) and then recovered after 4 weeks of recovery acclimation at a normal temperature (22°C). No significant variation in the expression of *Hsp60* mRNA was found (Fig. 5).

### Gonad development and reproductive endocrine responses to chronic thermal stress

**Fig. 6.**
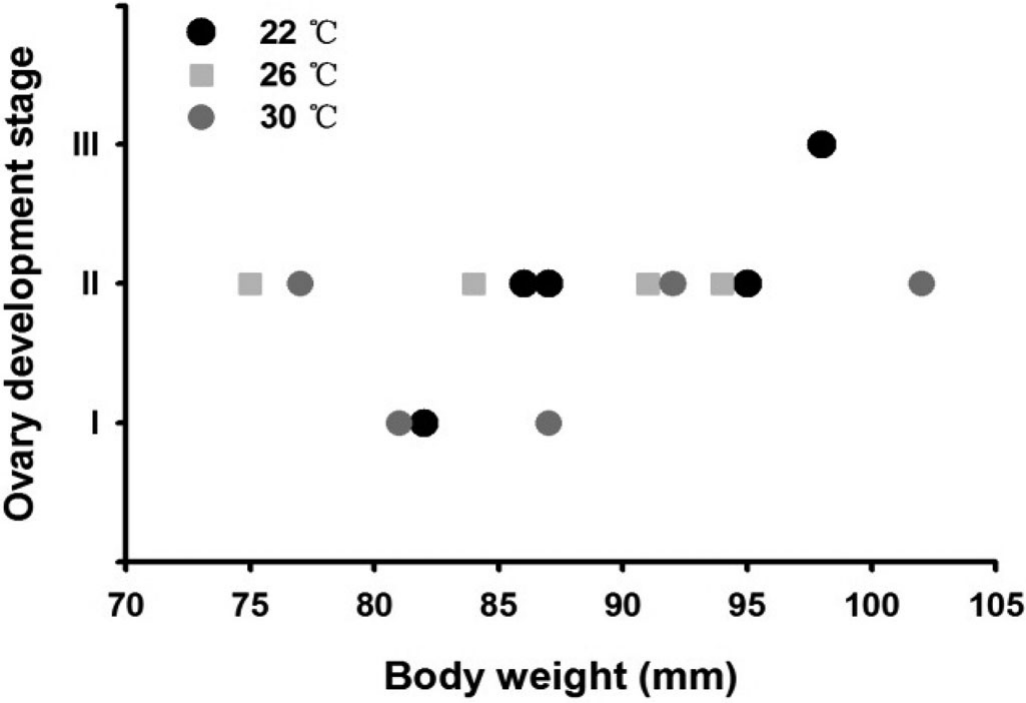
**Ovary development of *Hippocampus erectus* seahorses under different chronic thermal treatments.**

**Fig. 7.**
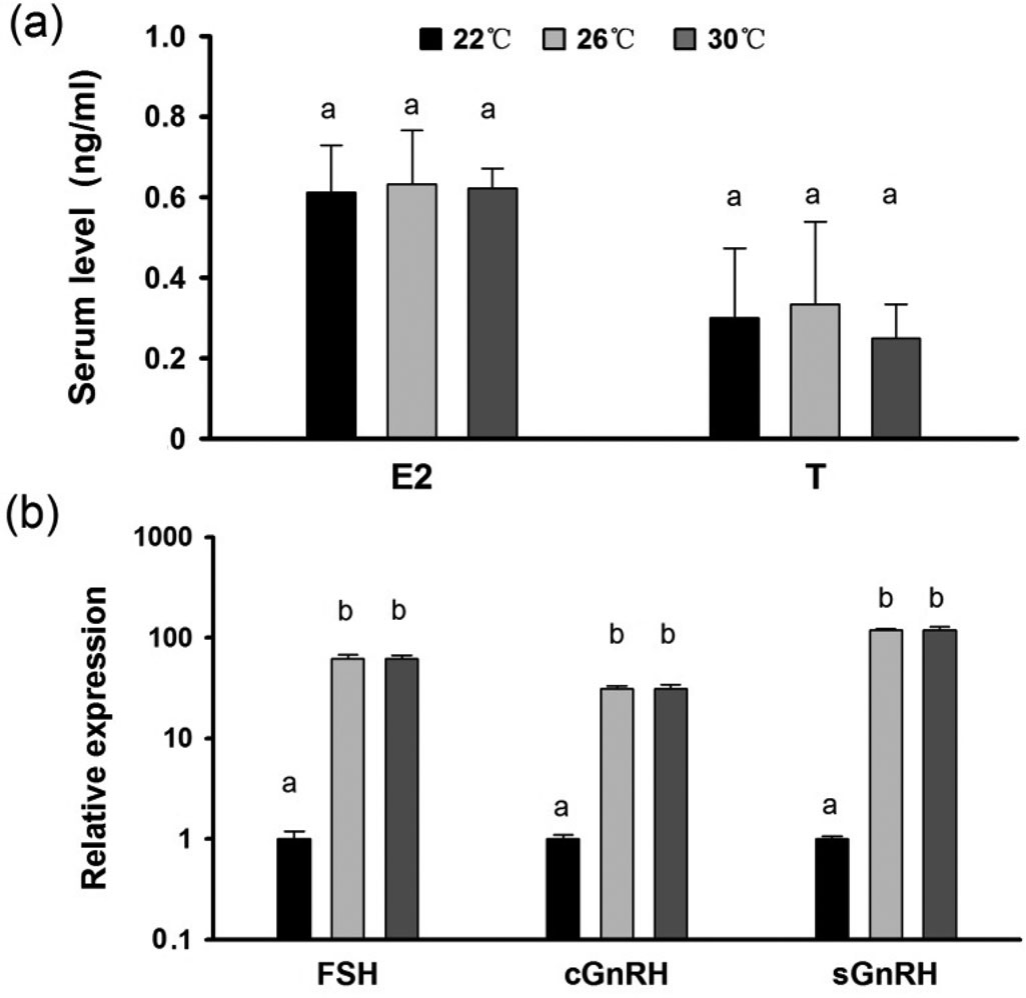
**Reproductive endocrine responses of seahorses in response to chronic thermal stress.** (A) Sex hormone levels. (B) Reproductive regulation-related genes in brain and pituitary gland in response to chronic thermal stress. Different letters indicate significant differences between different groups.

Most of the female seahorses had developed ovaries by Stage II, and no significant differences were found between the different treatments (Fig. 6, ANOVA, *F*=1.531, *P*=0.256). Additionally, no significant variation in estrogen (E_2_) and testosterone (T) serum levels was found (*P*>0.05, Fig. 7A). The 30°C treatment led to significant up-regulation of *cGnRH*, *sGnRH* and *FSH* gene expression in the seahorses’ brains compared with the 22°C seahorses (*P*<0.05), while no differences compared with the 26°C seahorses were found (Fig. 7B).

## DISCUSSION

Due to the potential responses of seahorses to temperature variations during inshore-offshore migration, the present study addressed the behavioral and physiological responses of adult seahorses to thermal stress, especially regarding the changes in the reproductive characteristics of male seahorses (Lin et al., 2006, 2007, 2010). Previous studies have mainly focused on swimming, breathing and feeding (Aurélio et al., 2013; Faleiro et al., 2015; Mascaró et al., 2016). Fish reproduction only occurs within a bounded temperature range and is assumed to be much more sensitive than other physiological processes (Pörtner and Farrell, 2008). However, few previous studies have focused on the effects of thermal stress on the seahorse reproductive system, especially regarding gonad development and the reproductive endocrine system, with the exception of some field survey data (Baum et al., 2003). Adult seahorses seasonally migrate into warm waters for breeding and might suffer from thermal stress during migration or within their breeding grounds (Curtis and Vincent, 2006; Qin et al., 2017).

As expected, the ventilation rate of the lined seahorses increased significantly under temperatures up to 30°C during the acute thermal stress experiment, in line with results obtained for *Hippocampus guttulatus* and *Hippocampus kuda* (Aurélio et al., 2013; Faleiro et al., 2015). The sharp up-regulation of *Hsp60* and *Hsp70* gene expression in the seahorses’ livers indicated that acute thermal stress could lead to serious cell damage (Iwama and Thomas, 1998). When exposed to 32°C thermal stress, the lined seahorses could not maintain normal aerobic respiration and died within a few hours, implying that extreme thermal events during migration could directly cause acute death in seahorses.

Acclimation can compensate for the effects of thermal variation in the environment on physiological functions and is an important mechanism whereby marine organisms cope with environmental temperature changes (Donelson et al., 2010). However, compared with the seahorses exposed to 26°C or 22°C, the 30°C seahorses showed a persistent increase in ventilation, a lower HSI index and higher mortality rates during the 4 weeks of thermal stress, indicating that migratory seahorses cannot adapt to 30°C thermal stress, even through acclimation. *Hsp* gene expression was up-regulated for several hours in seahorses suffering from thermal stress and then gradually decreased, but was still higher than in untreated seahorses after the 4 weeks of chronic thermal stress treatment, implying that the cell damage caused by thermal stress could not be healed over time through acclimation (Feder and Hofmann, 1999). Therefore, we predict that temperature could affect energy expenditure directly and cause the accumulation of various types of molecular and cellular damage. All of the deaths observed in the 30°C treatment occurred only 2 weeks later, suggesting that the seahorses’ thermal tolerance was time dependent.

Feeding is a primary factor affecting fish growth, and temperature increases within an appropriate range usually promote fish predation and digestion; however, excessive thermal stress causes metabolic disturbance and decreases feeding ability (Watkins, 1996). In the present study, increased feeding and defecation frequencies occurred in the 30°C treatment only during the first week, and the parameters then recovered in the following 3 weeks. The relatively stable *leptin* expression observed in the seahorses also suggested that thermal stress does not affect the appetite continually (Zhang et al., 2016). Previous studies have also confirmed that in juvenile *Hippocampus trimaculatus*, *H. erectus* and adult *H. guttulatus* food intake is not sensitive to thermal stress (Sheng et al., 2006; Aurélio et al., 2013). Given the higher metabolic rates of the 30°C seahorses compared with the 26°C seahorses, we assumed that thermal stress inhibited hepatic energy reserves and lead to the lower HSI in *H. erectus* by increasing energy consumption but not decreasing feeding efficiency. In contrast to the successive increases in the ventilation rate and higher *Hsp* gene expression, the feeding rates did not follow the increased metabolic rates with warming as expected; therefore, we assume that the seahorses may not have been able to meet the high energetic demands and may have accumulated cell damage during long-distance migration, leading to high rates of mortality.

Fish reproduction, including the reproductive endocrine system and gonad development, is sometimes more sensitive than other physiological processes to thermal stress (Ficke et al., 2007; Ziȩba et al., 2010; Zucchetta et al., 2012; Miranda et al., 2013). Given that most migratory seahorse species breed during the warm season (Teixeira and Musick, 2001; Baum et al., 2003; Qin et al., 2017), we predicted that seahorse reproduction would adapt to a higher thermal range and that thermal stress during migration might be tolerated in seahorses. The gonad is most readily damaged by heat treatments through the inhibition of gene expression and subsequent synthesis of different gonadal steroidogenic enzymes, independently of the brain–pituitary axis (Pankhurst et al., 2011). High temperatures can provoke a complete regression of the gonad in pejerrey *Odontesthes bonariensis* (Pankhurst et al., 1996; Miranda et al., 2013), depress T and 11-ketotestosterone (11-KT) levels in the plasma of pejerrey fish (Soria et al., 2008) and a sharp decrease in plasma E_2_ also appears to be a common response to elevated temperatures in female ﬁsh (Pankhurst and Munday, 2011). Although, a previous study on *H. kuda* showed that ovary development was significantly inhibited only upon exposure to a temperature of 32°C (Lin et al., 2006). In the present study, we did not find either gonad regression or serum hormone level changes under the 30°C thermal stress.

Excessive thermal stress can also disrupt the expression of fish reproductive endocrine genes in addition to the effects on gonad development (Elisio et al., 2012; Miranda et al., 2013; Wright et al., 2017). High water temperature can impair gonad development and block spawning, altering different components of the endocrine reproductive axis (Miranda et al., 2013). For example, high temperatures can affect the expression of GnRH in *Trichopodus trichopterus* (David and Degani, 2011; Levy et al., 2011), and *Fsh-b* expression levels at the pituitary is inhibited in females of *T. trichopterus* while being exposed to high temperatures (Levy et al., 2011). In this finding, similar gene expression levels of *Gnrh* and *Fsh* were observed in seahorse brains and pituitary glands between 26°C and 30°C, suggesting that thermal stress does not lead to a negative effect on the reproductive endocrine system.

The effects of climate change on ﬁsh and ﬁsheries are expected to be particularly pronounced in the shallow sea areas that are usually inhibited by seahorses (Brander, 2007). The range of *Hippocampus kelloggi* has been found to have extended southward from tropical zones into temperate zones, similar to those of other tropical marine species in recent years (Harasti, 2017). The increased frequency of prolonged heat waves associated with global warming might be sufficient to alter the geographical distribution of the lined seahorse in the southern Gulf of Mexico and the Caribbean (Mascaró et al., 2016). The present study confirmed that chronic thermal stress causes negative effects on basal metabolic energy dissipation and increases seahorse mortality rates. Considering that lined seahorses are widely distributed from tropical to temperate areas, global warming might impact the geographical populations of seahorses, perhaps in their population structure and genetic variation. Gene flow among different seahorse populations might be decreased greatly and lead to a much more significant gap in genetic structures between the tropical and temperate populations (Boehm et al., 2015). Moreover, global warming might also affect the environmental adaptation abilities of seahorses because of the variation of the candidate genes in seahorses and food chains in their habitats, which might directly influence the growth, survivorship and reproduction of seahorses in wild oceans.

## MATERIALS AND METHODS

### Collection and culture of seahorses

We collected 234 F3-generation breeding line seahorses *H. erectus* from the Shenzhen Seahorse Breeding Center (South China Sea Institute of Oceanology, Chinese Academy of Sciences, Guangdong Province, China). All seahorses were mature, with a body length ranging between 80 and 100 mm and aged between 5 and 6 months. Before the experiment began, the seahorses were placed in holding tanks (22°C) for 2 weeks for acclimation. The animals were maintained at a salinity of 25‰ under a 12 h dark: 12 h light cycle and were fed frozen *Mysis* shrimp at 2% seahorse body weight twice per day (09:00 and 16:00). The feces and residual diet were siphoned twice per day, and fresh artificial saltwater was added to the systems after cleaning. Each holding tank was supplied with an air stone and a plastic plant to provide a habitat for the seahorses. These animals were approved for use under IACUC #160413 through the Chinese Academy of Sciences.

### Acute temperature shift experiment

To evaluate short-term thermal stress on migratory seahorses, 180 seahorses were transferred from holding tanks (22°C) into pre-heated tanks at 22°C, 26°C, 30°C and 32°C for durations of 2, 6 and 10 h. Temperatures between 22°C and 26°C have been reported to be the optimal thermal range for lined seahorses (Lin et al., 2009; Mascaró et al., 2016). Seahorses often encounter a temperature of 30°C during their migration and breeding seasons, and 32°C is considered an extreme high temperature caused by heat wave events (Cook et al., 1998). Three replicate tanks were set up for each treatment, and six seahorses were placed in each tank (20 cm×30 cm×30 cm, 16 l). The number of opercular movements per minute (opening and closing of the gill covering) was recorded 1 h before the end of each treatment. Finally, all of the seahorses were sampled and dissected. Their brains (including pituitary glands) and livers were placed in individually labeled centrifuge tubes and immediately flash frozen with liquid nitrogen for molecular analysis.

### Chronic temperature stress experiment

To evaluate the effect of chronic thermal stress on seahorses during migration, a 4-week chronic thermal stress experiment was conducted from July to August 2016. Nine tanks served as housing for the chronic temperature treatments, with three tanks being maintained at each of the following three temperatures: 22°C, 26°C and 30°C. Twelve seahorses were placed in each tank. After a period of chronic thermal stress, half of the seahorses were sampled, while the others were transferred to a normal temperature (22°C) for an additional 28 days of acclimation before sampling.

Seahorse swimming was observed and recorded twice daily before feeding in the chronic treatment. Three swimming behavior categories were defined under a simple system of descriptive behavior (Qin et al., 2014). ‘Hitching’ was used to describe any seahorse whose tail was wrapped around a substrate exhibiting little body movement, suggesting the relaxed state of a non-stressed seahorse. ‘Swimming’ was used to describe any seahorse that was actively moving around the tank without making contact with any substrate. This behavior requires energy expenditure on the part of the seahorse and could indicate hunting or escape. ‘Resting’ was used to describe any seahorse making contact with the bottom of the tank without its tail wrapped around any substrate. This behavior was often observed in visibly stressed and fatigued seahorses. The number of opercular movements per minute was recorded twice per day to evaluate the basal metabolic level (Qin et al., 2014). The feeding frequency, deﬁned by the number of successfully preyed-upon *Mysis* per seahorse in 5 min, was recorded twice per day during feeding. In addition, the frequency of defecation, defined as number of seahorse fecal particles on the tank bottom, was counted early every morning. The variation of the defecation frequency was chosen to reflect the variation of the amount of feeding.

Finally, all of the seahorses were sampled and dissected and blood was collected for hormone detection. The ovary development stage and the HSI were recorded. Brain and liver tissues were placed in individually labeled centrifuge tubes and immediately flash frozen with liquid nitrogen. The wet weight and body height were also measured at the beginning of the experiment and at each sampling time, and growth rates were calculated. Dead seahorses were recorded every day and the mortality rate was calculated.

### RNA analysis

Total RNA was isolated from the frozen tissue samples using the TRIzol reagent (Invitrogen). The putative *Hsp60*, *Hsp70*, *leptin-α, leptin* *receptor*, *FSH*, *sGnRH*, and *cGnRH* cDNAs were identified from seahorse genomic data (Lin et al., 2016). The housekeeping gene β-actin was screened using Polymerase Chain Reaction (PCR) in tandem in the same samples to verify the integrity of the cDNA template across tissues. The PCR primers were designed based on the putative gene-coding sequences using Primer Premier 5.0 Software (Premier Biosoft, Palo Alto, CA, USA) (Table S1). All amplification reactions performed in the present study were carried out using the following PCR cycling parameters: denaturation at 94°C for 3 min, followed by 35 cycles at 94°C for 20 s, 52–56°C for 20 s and 72°C for 90 s. The reaction was terminated after an extension step of 10 min at 72°C. The expression level of each target gene, analyzed via real-time quantitative PCR (qPCR), was determined using the comparative quantiﬁcation method 2 ^–ΔΔCT^ (Livak and Schmittgen, 2001).

### Plasma hormones

After euthanasia with 0.2 mg/l MS-222 for 2 min, blood was collected by severing the tails of the seahorses. Serum was separated via centrifugation for 10 min at 3000 ***g***. Steroid concentrations (T, 11-KT and estrogen E_2_) were measured spectrophotometrically in diluted plasma using a radioimmunoassay kit (Beifang Ltd, Beijing, China).

### Data analysis

Statistical analyses were conducted using the software SPSS 19.0 (IBM) and Sigma PLOT 10.0 (Systat Software, New York, USA). One-way analysis of variance (ANOVA) was employed to assess the differences in swimming performance, ventilation frequency, feeding frequency, gene expression, and the growth and survival rates of the seahorses among the treatments, with a significance level of 0.05. If ANOVA indicated that the effects were signiﬁcant, comparisons between the different means were performed using the post hoc least significant differences (LSD) test.

## Supplementary Material

Supplementary information

First Person interview
