# Supplementary material for: Temperature-induced physiological stress and reproductive characteristics of the migratory seahorse Hippocampus erectus during a thermal stress simulation
Source: Biol Open. 2018 May 15;7(6):bio032888. doi: 10.1242/bio.032888 (PMC6031341; doi:10.1242/bio.032888)
Supplement: Supplementary information [file biolopen-7-032888-s1.pdf]

Table S1. Nucleotide sequences used in qPCR assays with the lined seahorse *Hippocampus erectus*

| Gene | Primer | 5′-3′ sequence |
|---|---|---|
| *β-actin* | Actin-F | GGGACCTGACTGACTACCTC |
| | Actin-R | TCATACTCCTGCTTGCTGAT |
| *Hsp60* | HSP60F3 | CGCCTTTCCAAGCCAGTC |
| | HSP60R3 | TGTAACCACGGTCAAACTTCAT |
| *Hsp70* | HSP70F4 | ATGGTGTCAGCGAAAGGC |
| | HSP70R4 | CCGTGTTGCTCGGGTTC |
| *Leptin receptor* | lepr qF | CCTACAACTCAGTGGAAGAAC |
| | lepr qR | TGTACGGTGCAGTCCTGAAC |
| *Leptin* | lepa qF | CGACAAGCTCATCTCAGACTC |
| | lepa qR | AGGATGTCCTTGACTCTGACC |
| *FSH* | FSHqF2 | ATGCAGCTAGTTGTCATGAC |
| | FSHqR2 | TGTTTCACTTTGTAGGTCCAG |
| *cGnRH* | cGnRH qF | GTTGAGTCCGACACCTTTGG |
| | cGnRH qR | GCCTCCAGGATACCAACCAT |
| *sGnRH* | sGnRH qF | CCTTGCGTAGCTGAGATGGAG |
| | sGnRH qR | TACATTGTATGGTCGACGTCTC |

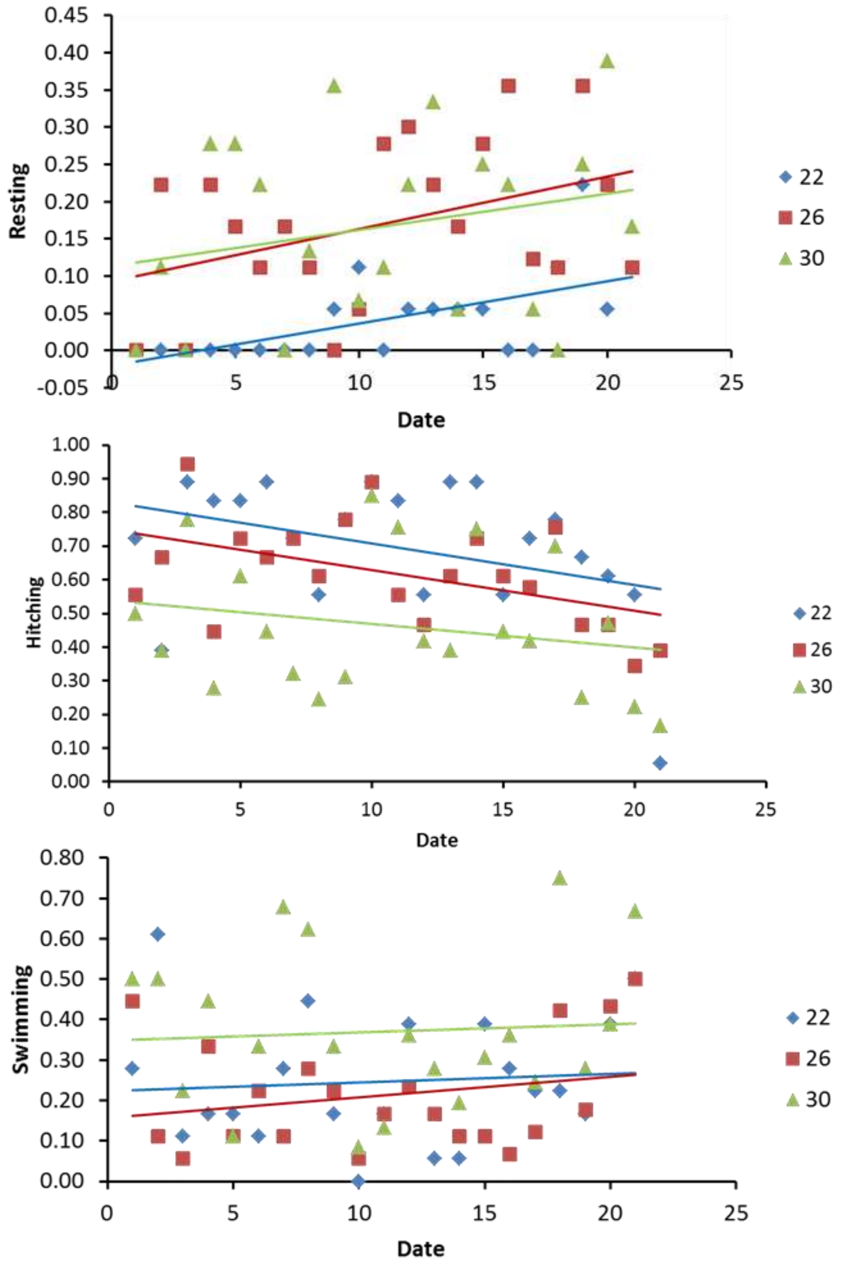


Fig. S1 Behavior of all seahorses throughout the course of chronic treatment (n=18 seahorses per temperature).

Behavioral categories include hitching (tail attached to a substrate), swimming (body moving freely in the water column), and resting (body making contact with bottom of tank but not attached to any substrate).

Biology Open • Supplementary information
