# Supplementary material for: Temperature-induced physiological stress and reproductive characteristics of the migratory seahorse Hippocampus erectus during a thermal stress simulation
Source: Biol Open. 2018 May 15;7(6):bio032888. doi: 10.1242/bio.032888 (PMC6031341; doi:10.1242/bio.032888)
Supplement: First Person interview [file biolopen-7-032888-s2.pdf]

© 2018. Published by The Company of Biologists Ltd | Biology Open (2018); doi:10.1242/bio.032888: Supplementary information

FIRST PERSON

First person – Geng Qin

First Person is a series of interviews with the first authors of a selection of papers published in Biology Open, helping early-career researchers promote themselves alongside their papers. Geng Qin is first author on 'Temperature-induced physiological stress and reproductive characteristics of the migratory seahorse *Hippocampus erectus* during a thermal stress simulation', published in BiO. Geng is a research assistant professor in the lab of Qiang Lin at South China Sea Institute of Oceanography, China, investigating the resources, biogeography and ecology of the seahorse.

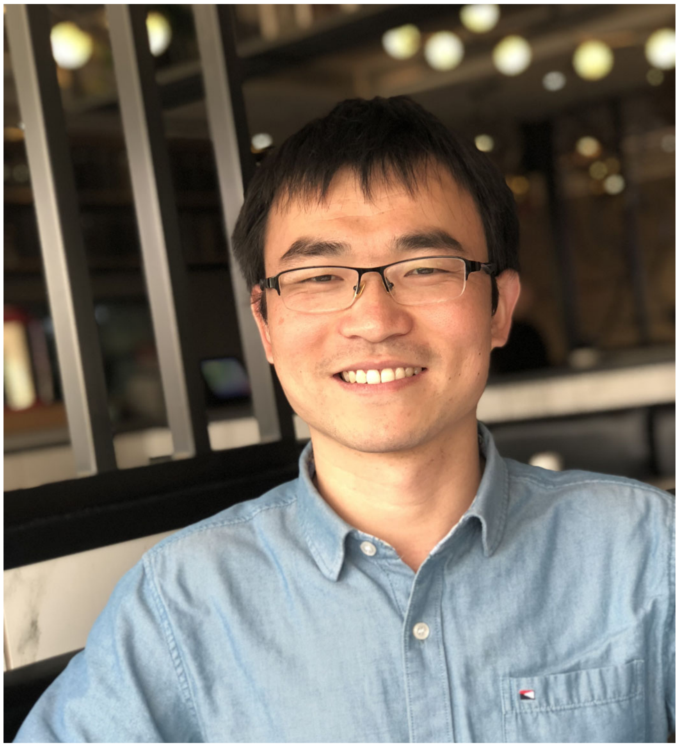

Geng Qin

What is your scientific background and the general focus of your lab?

My academic background ranges from marine biology to ecology, and I mainly focus on the population structure and environmental adaptation of seahorses. Currently, I am interested in seahorse migration and dispersal throughout the global oceans. I was lucky to join Prof. Qiang Lin's lab in 2009, as this team targets a wide range of research fields in biogeography, ecology and evolution, and conservation of the teleost family Syngnathidae, including seahorses, pipefish, pipehorses and seadragons.

How would you explain the main findings of your paper to non-scientific family and friends?

The seahorse belongs to a unique genus among marine fish, with some amazing features such as specialized morphology, prehensile tails and male pregnancy. However, the life history of seahorses in wild oceans is still unclear. Previously, we identified the existence of seasonal migration among seahorse populations. In this new study, the behavioral and physiological responses of seahorses under different thermal stress were studied. The results suggest that seahorses might experience massive mortality due to excessively high basal metabolic rates and stress damage during migration. Our findings indicate that the population sizes of some animals in the oceans might vary seasonally along the shore, and we need to protect them during their reproductive seasons so as to maintain stable resources for these species.

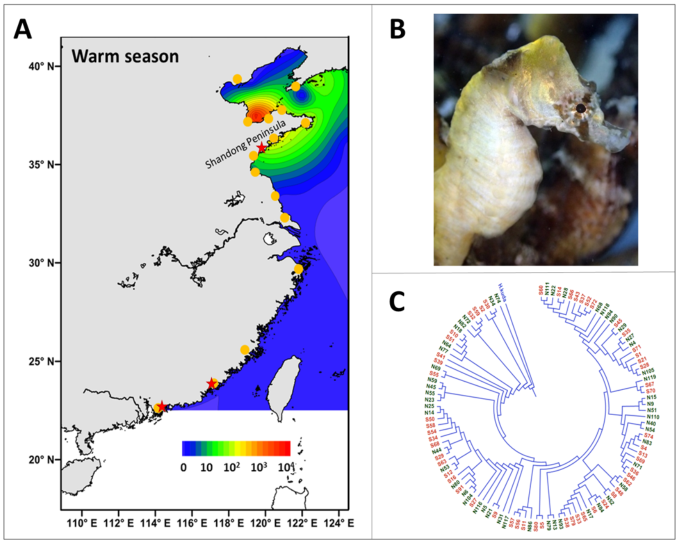

Seahorses migrate seasonally into shallow waters for breeding.

What are the potential implications of these results for your field of research?

This study is the first to systematically report the effects of seawater temperature changes on the physiological and behavioral responses of seahorses. The findings hold the key to understanding the impact of these responses on seahorse distribution and migration. Moreover, combined with ocean temperature data, we might be able to predict seahorse population fluctuations and habitat change in the future. In addition, this finding also provides potential data for the seahorse aquaculture and conservation.

What has surprised you the most while conducting your research?

The seahorse is specialized for male pregnancy through a male brood pouch. Our previous studies found that seahorses could migrate into shallow water areas seasonally for breeding, although they are really poor swimmers. While conducting this

Geng Qin's contact details: South China Sea Institute of Oceanology, Chinese Academy of Sciences, 164 Xingangxi Rd, Haizhu District, Guangzhou 510301, China.
E-mail: qingeng@scsio.ac.cn

study, one of our group findings was that some key genes play essential roles during seahorse reproduction. Thus, we decided to investigate whether expression of these genes varied under different environmental conditions.

"With the rapid development of genomics and bioinformatics analysis, we can now investigate population genetics and evolutionary ecology using the latest techniques."

What, in your opinion, are some of the greatest achievements in your field and how has this influenced your research?

With the rapid development of genomics and bioinformatics analysis, we can now investigate population genetics and evolutionary ecology using the latest techniques. For example, seahorses have a small body size and a low distribution density, but through sampling a few seahorse specimens we can obtain information about their genetic makeup and what kind of events led to their current state of evolutionary progress. I believe that these new techniques and novel ideas in marine biology will reveal a number of new findings very soon.

What's next for you?

A combination of environmental changes and heavy trade in seahorses for traditional Chinese medicine have led to a great decline in stocks of wild seahorses, especially in the Southeast Asian region. My research group and I hope to find effective ways to protect this mystical animal. Of course, some scientific research is very necessary for this work, and we will continue this charming job in the future.

Reference

Qin, G., Johnson, C., Zhang, Y., Zhang, H., Yin, J., Miller, G., Turingan, R. G., Guisbert, E. and Lin, Q. (2018). Temperature-induced physiological stress and reproductive characteristics of the migratory seahorse *Hippocampus erectus* during a thermal stress simulation. *Biol. Open* **7**: bio032888.
